# Aerogel/Hollow Glass Microsphere-Filled Styrene–Acrylic Composite Thermal-Insulating Coating for Architectural Decoration and Its Fabrication Mechanism

**DOI:** 10.3390/ma19204301

**Published:** 2026-10-13

**Authors:** Yanqing Cui, Qianhong Diao, Xian Liang

**Affiliations:** 1 School of Art Design, Hangzhou Polytechnic, Hangzhou 311402, China; cuiyq@hzpt.edu.cn; 2 Quzhou Housing and Urban-Rural Development Bureau, Quzhou 324000, China; 3 College of Civil Engineering and Architecture, Quzhou University, Quzhou 324000, China

**Keywords:** silica aerogel, hollow glass microspheres, hierarchical porous structure, synergistic thermal insulation, thermal-insulating coating, building energy efficiency

## Abstract

Hydrophobic silica aerogel was synthesized from a water-glass precursor via CO_2_-mediated carbonation. A water-based dispersion strategy was adopted to incorporate silica aerogel and hollow glass microspheres (HGMs) as dual insulating fillers into styrene–acrylic emulsion, yielding composite architectural coatings with hierarchically porous architecture. The impacts of filler loading and mass ratio on thermal-insulation performance were systematically explored. The optimized composite coating delivers a low thermal conductivity of 0.035 W/(m·K) and retains good thermal stability up to 350 °C. The outstanding thermal-insulating capability originates from the synergistic effect between macropores of hollow glass microspheres and nanopores of silica aerogel. Aerogel nanoparticles fill inter-particle voids among stacked hollow glass microspheres, break thermal-bridge conduction pathways, and suppress heat transfer by virtue of the intrinsically ultralow thermal conductivity of aerogel. Such structural features effectively mitigate both solid heat conduction and internal convective heat transfer within the coating matrix. This work establishes a facile, scalable route to fabricate gel-derived hierarchically porous thermal-insulating materials, showing promising applicability for functional decorative coatings in building energy-saving scenarios.

## 1. Introduction

Thermal insulation materials play a critically important role in reducing building energy consumption, improving the operational efficiency of industrial equipment, and promoting green and low-carbon development. With the intensification of the global energy crisis, the development of high-performance thermal insulation materials has become a research hotspot in the field of materials science [1,2,3,4]. However, traditional thermal insulation materials (such as asbestos, glass fiber, and expanded perlite) have inherent limitations, including relatively high thermal conductivity, high density, and difficulty in processing irregular surfaces, which make them increasingly unable to meet the demand for efficient, lightweight, and environmentally friendly thermal insulation materials in modern construction and industrial sectors [5,6,7,8,9]. Specifically, high thermal conductivity leads to unsatisfactory insulation performance and increased energy loss; high density results in greater material self-weight, which not only raises transportation costs but also imposes higher requirements on load-bearing structures; and difficulty in processing irregular surfaces limits their application adaptability on complex-shaped equipment or curved structures. These problems not only compromise the thermal insulation effect but also increase material costs and construction difficulties. Therefore, there is an urgent need to develop novel green thermal-insulating coatings suitable for architectural decoration scenarios to overcome the numerous shortcomings of existing materials, meet the application requirements of building exterior wall decorative coatings, achieve more efficient energy utilization, and promote the sustainable development of the ecological environment [10,11,12].

Based on the ultra-low thermal conductivity and excellent hydrophobic properties of aerogel [13,14,15,16,17,18,19,20], to overcome the aforementioned limitations of traditional thermal insulation materials, researchers have focused on developing novel composite thermal insulation coatings based on silica aerogel [21,22,23]. The current preparation methods mainly include the dispersion method, solid-state method, microemulsion method, and antisolvent method [24,25,26,27,28,29,30]. Among these, the dispersion method has become the most commonly used technical route due to its simple process, strong filler adaptability, and good compatibility with waterborne emulsions. Di et al. [31] employed the dispersion method to blend aerogel and hollow glass microspheres into a waterborne acrylic resin. When the mass ratio of the two fillers reached 1:1, the resulting coating exhibited a thermal conductivity as low as 0.050 W/(m·K) and a bonding strength as high as 1024 kPa. He et al. [4] used styrene–acrylic emulsion as the film-forming material, incorporating hollow glass microspheres and other fillers, and achieved a surface temperature difference of 163 °C with a coating thickness of 4 mm. In comparison, the solid-state method [32] suffers from high energy consumption and low efficiency; the microemulsion method [33] enables nanoscale uniform mixing but faces challenges in system stability control; the antisolvent method [34] offers high efficiency but requires stringent process conditions [35]. Based on the above comparison, the dispersion method is simple to operate, easy to scale up, and exhibits good compatibility with styrene–acrylic emulsion. Therefore, this study adopts mechanical stirring dispersion to blend aerogel and hollow glass microspheres into styrene–acrylic emulsion, aiming to investigate the effects of filler ratio and dispersion process on the thermal insulation and mechanical properties of the resulting coatings.

Therefore, hydrophobic silica aerogel was prepared using water glass as a raw material via CO_2_ carbonation, and the silica aerogel thermal insulation coating was synthesized by water dispersion technology. Using silica aerogel and hollow glass microspheres as the core thermal insulation components, a hierarchical porous structure was formed in the styrene–acrylic emulsion, resulting in a coating with excellent thermal insulation performance. When applied to the surface of an object, the coating exhibits a low thermal conductivity of 0.035 W/(m·K) and high thermal stability (below 350 °C). By comparing the addition amounts and ratios of silica aerogel and hollow glass microspheres in the thermal insulation coating, the effects of silica aerogel and hollow glass microspheres on the performance of the thermal insulation coating were investigated. The excellent thermal insulation performance of the composite coating originates from the synergistic insulation effect between the macropores of hollow glass microspheres and the micropores of silica aerogel. The interparticle gaps generated during the stacking of hollow glass microspheres in the coating are effectively filled by silica aerogel nanoparticles. This not only interrupts the thermal bridge conduction paths between the microspheres but also further suppresses heat transfer by utilizing the ultralow thermal conductivity of the aerogel itself, achieving synergistic thermal insulation between macropores and micropores. Through this hierarchical pore synergistic design, in which “macroporous hollow microsphere skeletons provide the main insulation cavities, and nanoporous aerogel fills the microsphere gaps and interrupts thermal bridges,” both heat conduction and convective heat transfer within the coating are significantly suppressed, thereby realizing excellent thermal insulation performance. The coating shows promising potential as a functional decorative coating material for energy-saving renovation of building envelopes.

## 2. Experimental Section

### 2.1. Preparation of Silica Aerogel

Liquid sodium silicate (water glass, Na_2_O·nSiO_2_, SiO_2_ content ≈ 26 wt.%, modulus ≈ 3.3) was purchased from Zhejiang Jiashan Chemical Co., Ltd., Jiaxing, China. Deionized water (resistivity ≥ 18.2 MΩ·cm) was used throughout. Carbon dioxide (CO_2_, purity ≥ 99.9%) was supplied by Hangzhou Jingong Gas Co., Ltd., Hangzhou, China. Ethanol (analytical reagent, ≥ 99.7%), n-hexane (analytical reagent, ≥97.0%), and trimethylchlorosilane (TMCS, ≥99.0%) were obtained from Sinopharm Chemical Reagent Co., Ltd., Shanghai, China. All reagents were used as received without further purification.

Liquid sodium silicate and deionized water were mixed at a mass ratio of 1:4 (sodium silicate concentration 90 g/L) in a 5 L stainless-steel reactor with mechanical stirring at 300 r/min for 10 min. CO_2_ gas (≥99.9%) was then continuously introduced at a flow rate of 1.0 L/min. The reactor pressure was adjusted to 0.3 MPa, and the temperature was maintained at 25 ± 2 °C by a water bath. The mixture was stirred at 300 r/min under 0.3 MPa for 60 min to obtain a wet sol. After the reaction, the gas flow was stopped, and the mixture was allowed to stand at 25 °C for 30 min until gelation occurred. The resulting wet gel was aged in the mother liquor at 25 °C for 24 h, then washed with deionized water (gel:water = 1:5, *v*/*v*) under vacuum filtration three times to remove inorganic salt ions. The water gel was subjected to solvent exchange with ethanol (gel:ethanol = 1:5, *v*/*v*) twice and with n-hexane (gel:n-hexane = 1:5, *v*/*v*) twice, each for 12 h at 25 °C. For hydrophobic modification, the solvent-exchanged gel was mixed with a TMCS/n-hexane solution at a volume ratio of 1:10 and reacted at 25 °C for 24 h. After modification, the gel was filtered under vacuum and dried following a stepwise program (60 °C→90 °C→120 °C, 2 h at each stage) to obtain the hydrophobic silica aerogel.

### 2.2. Preparation of Aerogel Insulation Coating

The dispersant (polyacrylic acid sodium salt, 40 wt.% aqueous solution), wetting agent (non-ionic alkylphenol ethoxylate), and defoamer (mineral-oil-based) were supplied by BASF (Shanghai, China) Co., Ltd. Rutile titanium dioxide (TiO_2_, ≥93 wt.%), zirconium dioxide (ZrO_2_, ≥99.0%), and aluminum silicate (fibrous, Al_2_O_3_·SiO_2_, ≥99.0%) were purchased from Sinopharm Chemical Reagent Co., Ltd., Shanghai, China. The styrene–acrylic emulsion (solid content 48 ± 1 wt.%, Tg ≈ 25 °C, pH 7.5–8.5) was obtained from Dow Chemical (Shanghai, China) Co., Ltd. Hollow glass microspheres (HGMs, types S38, S60, and K46; density 0.38–0.60 g/cm^3^) were supplied by 3M China Ltd. (Shanghai, China). The coalescing agent (2,2,4-trimethyl-1,3-pentanediol monoisobutyrate) and associative thickener (hydrophobically modified ethoxylated urethane, HEUR) were purchased from Elementis Specialties (London, UK) and Ashland Inc. (Covington, KY, USA), respectively.

Deionized water (63.5 g), dispersant (1.0 g), wetting agent (0.5 g), and defoamer (0.5 g) were mixed and stirred at 500–600 r/min for 10 min. Silica aerogel (2.5 g), rutile TiO_2_ (1.0 g), ZrO_2_ (1.0 g), and aluminum silicate (1.0 g) were then added, and the mixture was stirred at 1000–1200 r/min for 1 h. Subsequently, styrene–acrylic emulsion (25.0 g) and hollow glass microspheres (S38, 2.5 g) were added, and the mixture was stirred at 400 r/min for 40 min. Finally, defoamer (0.3 g), coalescing agent (0.7 g), and associative thickener (0.5 g) were added, and the mixture was stirred at 500 r/min for 5–10 min to obtain the thermal-insulating slurry. The total mass was 100.0 g, corresponding to 2.5 wt.% aerogel, 2.5 wt.% HGM, 1.0 wt.% TiO_2_, 1.0 wt.% ZrO_2_, 1.0 wt.% aluminum silicate, and 25.0 wt.% styrene–acrylic emulsion.

The slurry was applied onto iron plates or stone boards with a doctor blade (wet-film thickness 1.0 mm) and dried at 25 ± 2 °C and 50 ± 5% RH for 7 days. The dry-film thickness was 0.6 ± 0.05 mm. For thermal conductivity measurement, the slurry was cast into 100 mm × 100 mm × 10 mm molds and dried under the same conditions.

### 2.3. Materials Characterization

The aerogel was characterized by SEM (Hitachi S-4700, Hitachi, Tokyo, Japan). An ASAP2020HD88 volumetric sorption analyzer (Micromeritics Instrument Corporation, Norcross, GA, USA) was used to measure the Brunauer–Emmett–Teller (BET) specific surface areas and pore distribution. X-ray diffraction (XRD) patterns were recorded with a PANalytical X’Pert PRO powder diffractometer (PANalytical, Almelo, The Netherlands) using Cu K_α_ radiation (λ = 0.1541 nm). The working voltage was 40 kV and the working current was 40 mA. The patterns were collected across a 2θ range from 10° to 80° with a step size of 0.05°. Fourier Transform Infrared (FTIR) spectra were recorded with a Nicolet iS 50 (Thermo Fisher Scientific, Waltham, MA, USA), collected over a wavenumber range from 500 cm^−1^ to 4000 cm^−1^.

Thermogravimetric analysis (TGA). Thermogravimetric curves of the aerogel samples and the composite coatings were recorded on a simultaneous thermal analyzer (NETZSCH STA 449F3, NETZSCH, Selb, Germany). Approximately 8–10 mg of each sample was placed in an alumina crucible (Al_2_O_3_, 85 μL) and heated from 30 °C to 800 °C at a heating rate of 10 °C·min^−1^ under a flowing N_2_ atmosphere (50 mL·min^−1^). An empty alumina crucible was used as the reference. The baseline was corrected with a blank run under identical conditions before each measurement. The reported TG curves are representative of at least two independent runs, and the temperature reproducibility was within ± 2 °C.

Contact angle measurement. The surface hydrophobicity of the aerogel samples was evaluated by a contact angle goniometer (Dataphysics OCA 20, Dataphysics Instruments GmbH, Filderstadt, Germany) using the sessile drop method. A deionized water droplet of 5 μL was deposited onto the flat aerogel surface with a microsyringe. The contact angle was read 10 s after droplet deposition to allow the droplet to reach a quasi-equilibrium state. At least five different positions on each sample were measured, and the average value was reported. All measurements were performed at room temperature (25 ± 1 °C) and a relative humidity of 50 ± 5%. The measurement uncertainty was within ±2°.

N_2_ adsorption–desorption measurement. The specific surface area and pore size distribution of the aerogel samples were determined by N_2_ adsorption–desorption at 77 K on a volumetric sorption analyzer (Micromeritics ASAP 2020HD88, Micromeritics Instrument Corporation, Norcross, GA, USA). Prior to measurement, each sample (~100 mg) was degassed under vacuum (10^−3^ Pa) at 120 °C for 6 h to remove physically adsorbed water and residual solvent. The specific surface area was calculated by the Brunauer–Emmett–Teller (BET) method in the relative pressure range of 0.05–0.30. The pore size distribution was derived from the desorption branch of the isotherm using the Barrett–Joyner–Halenda (BJH) model. The total pore volume was obtained at P/P_0_ ≈ 0.99.

Thermal conductivity. The thermal conductivity of the aerogel monoliths and coatings was measured by the steady-state guarded heat flow meter method using a DRPL-III thermal conductivity tester (Xiangyi Instrument Co., Ltd., Xiangtan, China). Coating specimens were cast into 100 mm × 100 mm × 10 mm plates, dried at ambient conditions for 7 days, and polished to parallel surfaces. The hot and cold plate temperatures were set at 35 °C and 15 °C, respectively (mean 25 °C, ΔT = 20 K). Steady state was defined as a heat flux variation of less than 1% over 30 min. Thermal grease was applied at the specimen–plate interfaces, and contact resistance was corrected by the two-thickness method. Each specimen was measured at least three times, with a relative standard deviation within ±3%.

Adhesion test. The adhesion of the coating to the substrate was evaluated by the cross-cut method according to GB/T 9286 using a cross-cut tester. A lattice pattern with six parallel cuts in each direction, spaced 2 mm apart, was made on the coated surface with a tungsten carbide blade. A pressure-sensitive adhesive tape was then applied over the lattice and pulled off at an angle of approximately 60°. The adhesion was rated according to the standard scale from 0 (no peeling) to 5 (severe peeling). At least three positions on each specimen were tested, and the average grade was reported.

Water resistance, light resistance, and pencil hardness tests. Water resistance was tested according to GB/T 1733 by immersing coated specimens in deionized water at 23 ± 2 °C for 96 h. Light resistance was evaluated by xenon-arc artificial weathering according to GB/T 1865, and the colour change was assessed visually according to GB/T 9761 using the grey scale. Pencil hardness was measured according to GB/T 6739-2022 using pencils from 9B to 9H under a load of 500 ± 5 g at a 45° angle.

## 3. Results and Discussion

### 3.1. Characterization of Silica Aerogel

**Figure 1:**
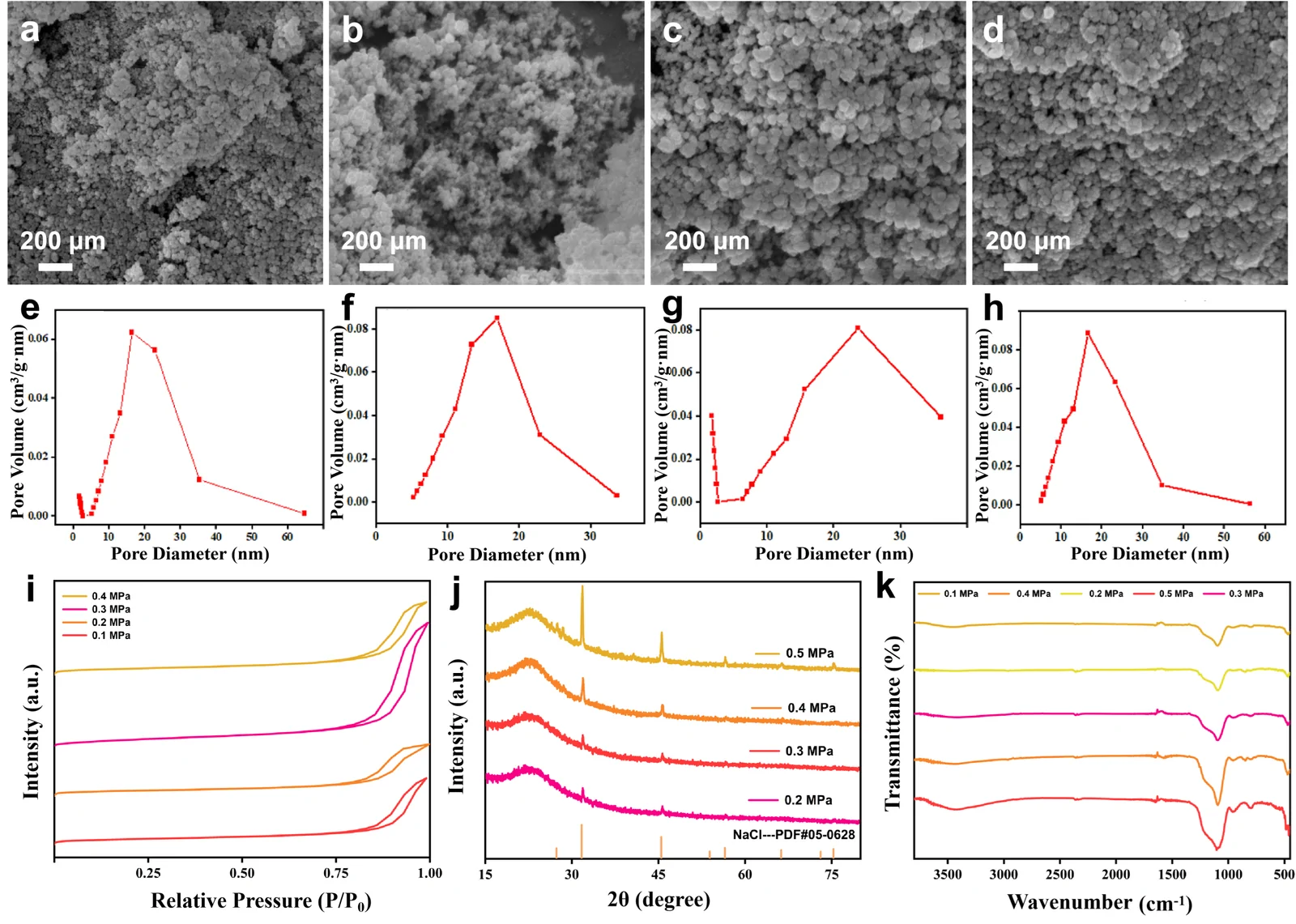
SEM images of silica aerogels prepared under different CO_2_ pressures: (**a**) 0.2 MPa, (**b**) 0.3 MPa, (**c**) 0.4 MPa, and (**d**) 0.5 MPa. Pore size distribution curves of silica aerogels prepared at different CO_2_ pressures: (**e**) 0.2 MPa, (**f**) 0.3 MPa, (**g**) 0.4 MPa, and (**h**) 0.5 MPa. (**i**) N_2_ adsorption–desorption isotherms, (**j**) XRD patterns, and (**k**) FTIR spectra of silica aerogels prepared under different CO_2_ pressures.

To investigate the effect of carbon dioxide pressure on the synthesis of silica aerogels, characterization was performed using SEM, BET, XRD, and FTIR. Figure 1 reveals the regulation pattern of carbon dioxide pressure on the microstructure of silica aerogels. All aerogel samples were subjected to solvent exchange and hydrophobic modification treatments, and were finally dried at ambient pressure to obtain silica aerogels. Figure 1a–d correspond to the SEM images of aerogel samples prepared under pressures ranging from 0.2 to 0.5 MPa, respectively. The SEM images show that all four groups of samples exhibit a typical three-dimensional porous network structure, which is formed by the interconnection of nano-sized particles, consistent with the structural characteristics of aerogels. By comparing the microstructures of aerogels prepared under different carbon dioxide pressures, it was found that carbon dioxide pressure plays a significant role in regulating the aerogel microstructure. The sample prepared at 0.3 MPa exhibits the most optimal three-dimensional network structure and abundant pore structure. It can be observed from Figure 1a that its three-dimensional network structure is less developed than that in Figure 1b, which is attributed to the relatively low carbon dioxide pressure; at lower pressures, the reaction between carbon dioxide and sodium silicate solution proceeds more slowly, which is unfavorable for the formation of extensive pore structures. When the carbon dioxide pressure is further increased, Figure 1c,d are obtained. As can be seen from the SEM images, compared with Figure 1b, the skeleton structure becomes coarser and the pore structure becomes correspondingly less abundant. This is because at higher carbon dioxide concentrations, the reaction between the carbon source and sodium silicate solution is more vigorous, resulting in a coarser gel skeleton. Therefore, the aerogel prepared at a carbon dioxide pressure of 0.3 MPa exhibits a favorable morphological structure.

To further investigate the specific surface area and pore size/pore volume distribution of the aerogels, N_2_ adsorption–desorption characterization was performed on aerogels prepared under different carbon dioxide pressures. Figure 1e–h show the pore size distribution curves of aerogels prepared under various CO_2_ pressures [36]. From these figures, it can be clearly observed that the pore sizes of aerogels prepared via the carbonation method are predominantly concentrated in the range of 10–30 nm, which is consistent with the typical pore size characteristics of mesoporous materials. Figure 1i presents the N_2_ adsorption–desorption isotherms of silica aerogels prepared under different CO_2_ pressures. It can be seen that all isotherms exhibit a similar trend with increasing relative pressure. The first inflection point appears at a relative pressure of around 0.8. Before this point, the curve is relatively flat, indicating that monolayer adsorption reaches its maximum at P/P_0_ ≈ 0.8. Beyond this relative pressure, the curve rises steeply, signifying the transition from monolayer to multilayer adsorption, with a sharp increase in adsorption volume, eventually forming an adsorption saturation plateau. According to the IUPAC classification of N_2_ adsorption–desorption isotherms, this type of isotherm belongs to Type IV. Furthermore, analysis of the specific surface area data reveals that, as shown in Table 1, the optimal aerogel sample exhibits a specific surface area of 531.4 m^2^/g. This is attributed to the fact that aerogels prepared via the carbonation method are porous materials with pore sizes predominantly distributed in the mesoporous range of 10–30 nm, which favors the formation of a high specific surface area. Carbon dioxide pressure has a significant influence on both the pore structure and specific surface area of the aerogels. Therefore, in practical synthesis, by adjusting parameters such as CO_2_ pressure, the pore structure and overall performance of the aerogels can be further optimized to meet the application requirements in various fields.

X-ray diffraction (XRD) was employed to analyze the crystalline phase composition and crystallinity of the silica aerogels. Figure 1j presents the XRD patterns of aerogels prepared under different CO_2_ pressures. Within the pressure range of 0.2–0.5 MPa, all samples exhibited a broad diffuse peak centered at approximately 2θ = 20°, indicative of the typical amorphous structure of silica aerogels, and the variation in CO_2_ pressure did not induce any crystalline phase transformation. Meanwhile, sharp diffraction peaks of varying intensities were observed at 31° and 45° in all samples, which were identified as the (200) and (220) planes of NaCl by comparison with the standard card (PDF #05-0628), revealing the presence of minor NaCl impurities in the as-prepared aerogels [37]. Notably, at a CO_2_ pressure of 0.3 MPa, the intensity of the NaCl diffraction peaks reached its minimum, suggesting the lowest residual Na^+^ content within the aerogel pores and indirectly indicating a more favorable pore structure that facilitates the displacement and removal of Na^+^. Fourier transform infrared spectroscopy (FTIR) was employed to verify the success of hydrophobic modification of aerogels prepared via the carbonation method. As shown in Figure 1k, the infrared spectra of aerogels prepared under different CO_2_ pressures all exhibited a strong and broad absorption band at 1061 cm^−1^, attributed to the asymmetric stretching vibration of Si-O-Si, which confirms the presence of the silica aerogel network structure and verifies the successful preparation of aerogels [38]. Meanwhile, the stretching vibration band of hydroxyl groups (-OH) near 3468 cm^−1^ was significantly diminished, while distinct Si–C stretching vibration bands appeared at 756 cm^−1^ and 843 cm^−1^, accompanied by a weak -CH_3_ symmetric deformation vibration at 1375 cm^−1^. These characteristic bands collectively indicate that TMCS was successfully grafted onto the aerogel surface, achieving effective hydrophobic modification. Furthermore, the CO_2_ pressure was found to exert a certain influence on the modification efficiency. When the pressure was raised to 0.5 MPa, the hydrophobic modification became insufficient, with some hydrophilic groups remaining unmodified. This phenomenon can be primarily attributed to the dense surface packing of the aerogel at excessively high pressure, which impeded effective contact between the modifying solution and the surface hydrophilic groups, thereby leading to incomplete modification.

### 3.2. Performance Analysis of Aerogels

**Figure 2:**
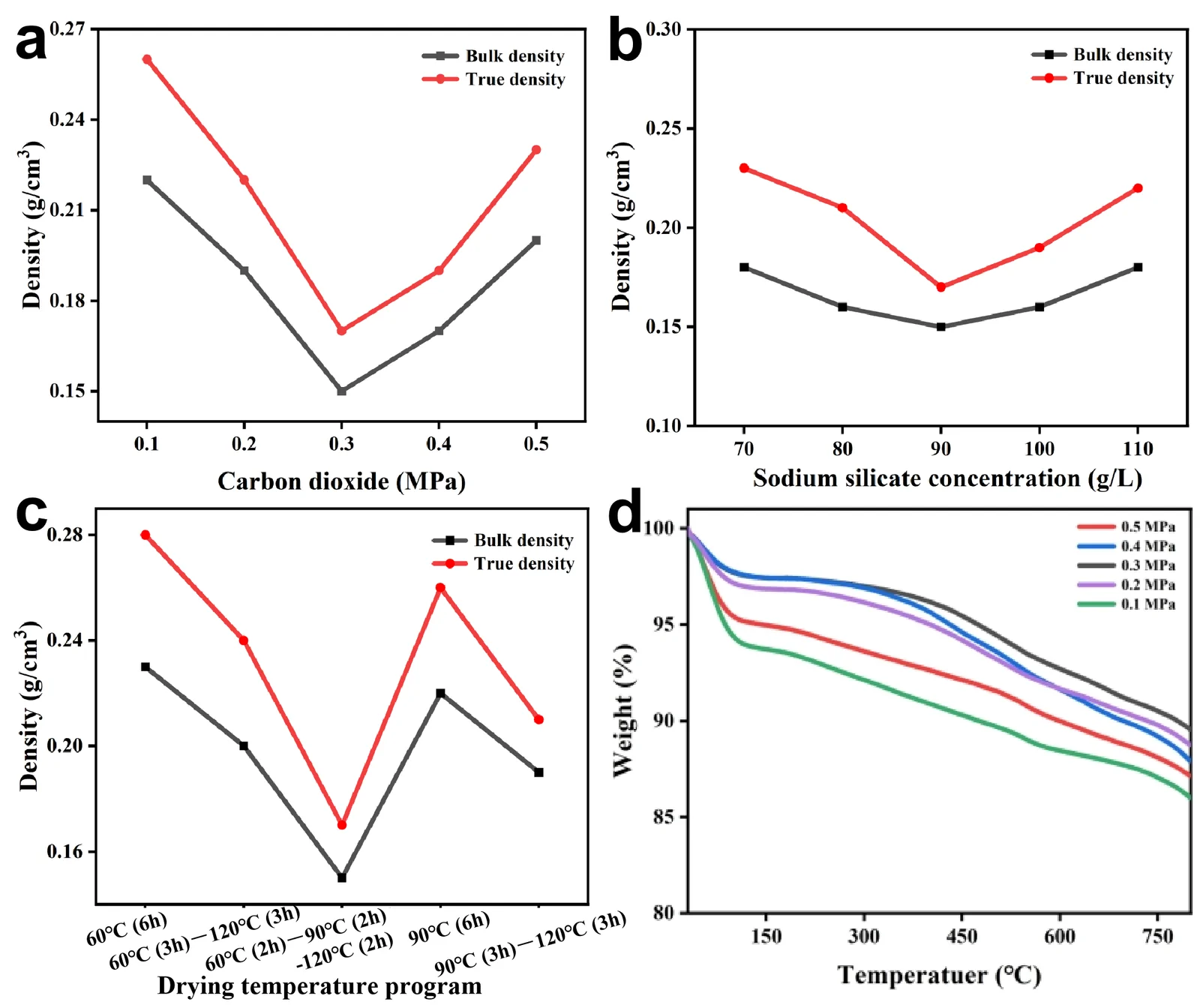
True density and bulk density of silica aerogels prepared under different conditions: (**a**) varying CO_2_ pressures, (**b**) different sodium silicate concentrations, and (**c**) various drying programs. (**d**) The thermogravimetric curve diagram of aerogels prepared under different carbon dioxide pressures.

To systematically investigate the physical properties of the silica aerogel, the bulk density and particle density of the aerogel samples prepared under varying conditions were first systematically measured. Density measurements were performed via multiple repeated tests, with the arithmetic mean calculated thereafter, so as to effectively minimize experimental errors and ensure the accuracy and reliability of the results. Initially, the bulk density and particle density of the aerogel samples obtained under different CO_2_ pressures were compared (Figure 2a). The bulk density of the silica aerogel was found to be lower than its true (skeletal) density. With increasing CO_2_ pressure, both the true density and the bulk density exhibited an initial decrease followed by a subsequent increase. This trend can be attributed to the fact that elevated CO_2_ pressure coarsens the skeletal structure of the silica aerogel, thereby enhancing its resistance to the surface tension of the liquid during drying and mitigating pore collapse caused by excessive surface tension. However, as the CO_2_ pressure continues to rise, the skeletal structure becomes excessively coarse, which impedes the effective exchange between the liquid within the pores and the ambient air during drying, consequently leading to increases in both the true density and the bulk density. In the preparation of silica aerogels, lightweight characteristics are regarded as a critical performance indicator; therefore, the density must be rigorously controlled to avoid excessive densification. Adjusting the CO_2_ pressure to 0.3 MPa proves beneficial for optimizing the pore structure and removing residual solvent, thereby ensuring the production of lightweight silica aerogels with superior overall performance. The densities of the samples obtained after reaction at different sodium silicate concentrations were compared. As shown in Figure 2b, the bulk density and true density of the as-prepared silica aerogels exhibited identical trends, yet the variation in bulk density was less pronounced than that in true density. At a sodium silicate concentration of 90 g/L, both the true density and bulk density of the silica aerogel reached their minimum values, namely 0.18 and 0.15 g/cm^3^, respectively. This observation can be rationalized as follows: at lower sodium silicate concentrations, the gel skeleton formed is rather fragile and thus collapses under the surface tension of the liquid during ambient-pressure drying, ultimately resulting in a higher density of the final product. Conversely, excessively high sodium silicate concentrations give rise to an overly coarse skeletal structure, which likewise leads to elevated density. Therefore, at a sodium silicate concentration of 90 g/L, the reaction between CO_2_ and sodium silicate proceeds more thoroughly, enabling the formation of a well-developed skeletal architecture, which in turn yields the lowest density among the prepared silica aerogels. The ambient-pressure drying temperature exerts a considerable influence on the density of the final product. As depicted in Figure 2c, both the bulk density and true density fluctuated markedly with variations in drying temperature. When the ambient-pressure drying temperature was set too low, the liquid within the pore channels could not be completely removed; the residual liquid, upon drying, occupied spaces that should otherwise have been occupied by gas or voids, thereby reducing the intrinsic volume of the aerogel and increasing its density. Conversely, when the drying temperature was raised directly to an excessively high level, the pore structure of the aerogel underwent collapse, which consequently compromised the product density and resulted in an increase thereof. In contrast, employing a stepwise temperature ramp (60 °C⟶90 °C⟶120 °C, 2 h at each stage) not only enabled efficient removal of the liquid from the pore channels but also prevented structural collapse, thereby yielding a product with lower density.

**Figure 3:**
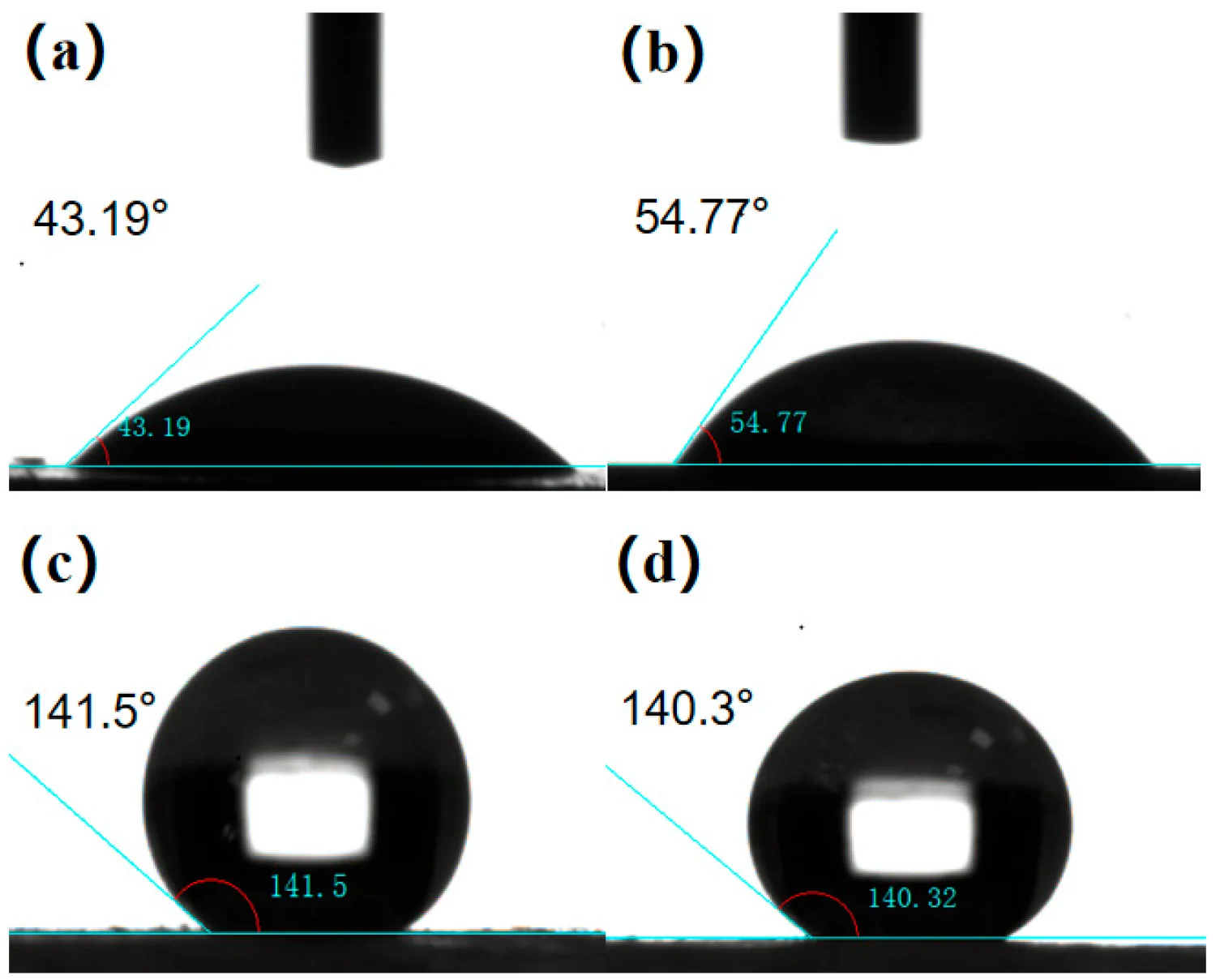
Comparison of pure water contact angles of materials before (**a**,**b**) and after (**c**,**d**) hydrophobic modification.

**Figure 4:**
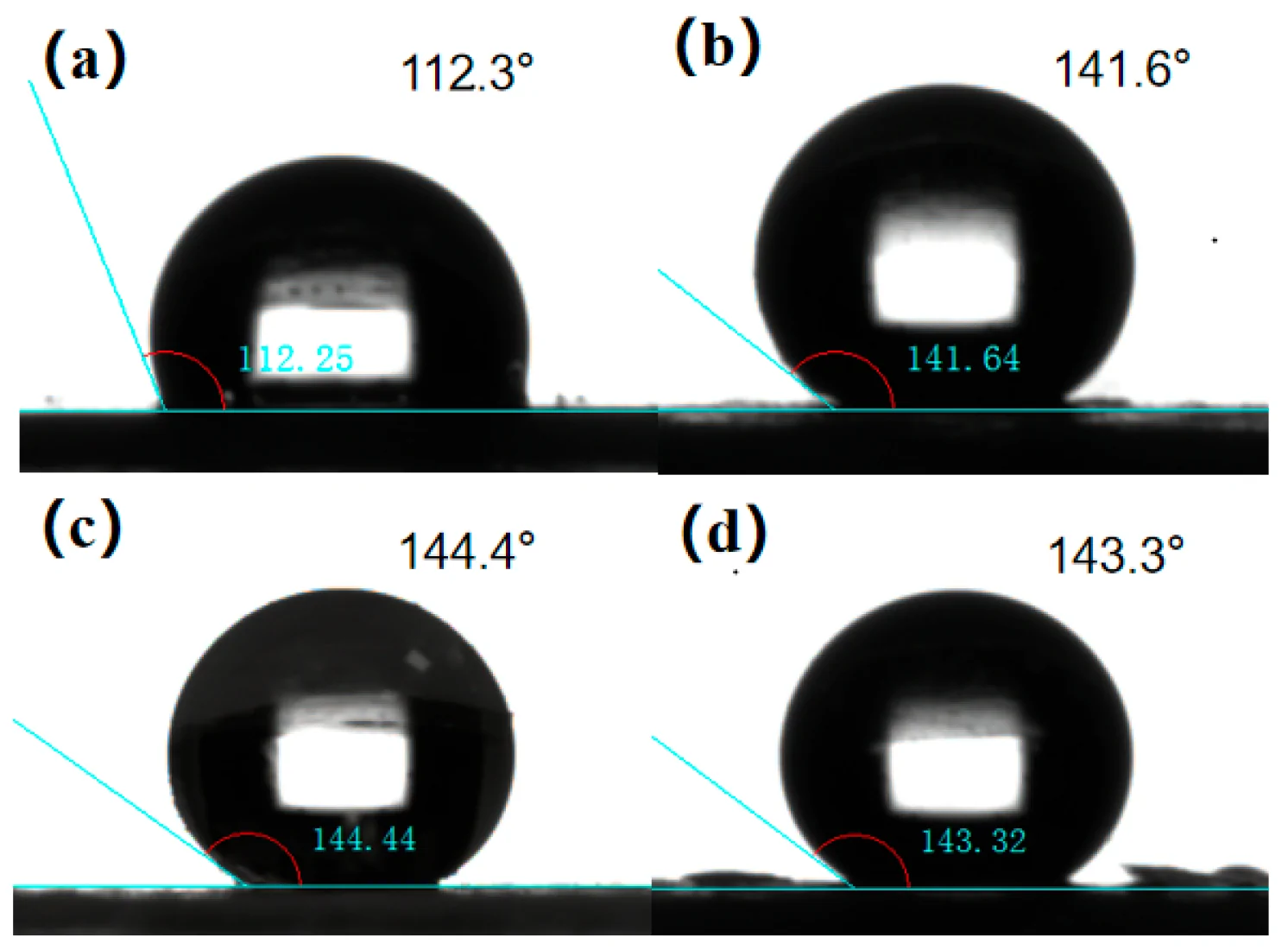
Pure water contact angles of aerogels prepared with different modification solution ratios: (**a**) 1:14; (**b**) 1:12; (**c**) 1:10; (**d**) 1:8.

Hydrophobic modification constitutes a crucial step in the preparation of silica aerogels. Hydrophobic silica aerogels are capable of maintaining stable performance in humid environments, resisting erosion by ambient moisture, and thereby enhancing the stability and durability of the material [39]. Initially, the pure water contact angles of the aerogel samples before and after hydrophobic modification were compared (Figure 3). As shown in Figure 3a,b, the aerogel without modifier treatment exhibited a pure water contact angle of approximately 50°, indicating no hydrophobic properties. In contrast, Figure 3c,d demonstrate that after hydrophobic modification with the modifying solution, the pure water contact angle of the aerogel exceeded 140°, conferring hydrophobic characteristics and approaching superhydrophobicity. These results indicate that surface modification with the modifying agent successfully grafted hydrophobic groups such as -CH_3_ onto the aerogel surface, thereby endowing the aerogel with hydrophobic properties. To investigate the effect of different modification solution ratios on the hydrophilic–hydrophobic properties of the resulting aerogels, pure water contact angle measurements were conducted on the aerogel samples. As shown in Figure 4, when aerogels were modified with the same volume of modification solution at varying ratios, the pure water contact angle remained relatively low at lower TMCS contents. With increasing TMCS content, the contact angle correspondingly increased. However, when the modification solution ratio reached 1:10, further increases in TMCS content resulted in virtually no change in the contact angle. This behavior can be rationalized as follows: at insufficient TMCS levels, the aerogel could not be fully modified, leading to a reduced contact angle; conversely, when the TMCS content was excessive, the surplus TMCS did not participate in the reaction, thereby causing the contact angle to plateau. Therefore, for hydrophobic modification, a modification solution ratio of 1:10 is recommended to obtain aerogel samples with relatively high hydrophobicity.

The thermal stability of the aerogel material was characterized by thermogravimetric analysis (TGA), as shown in Figure 2d. The results revealed that upon heating to 150 °C, the thermogravimetric (TG) curve exhibited a pronounced decline, which is attributed to the evaporation of water and other residual solvents within the aerogel pores. In conjunction with the microstructural characterization and density measurements, it was found that aerogels with a more abundant pore structure and lower density displayed a smaller decrease in the TG curve, indicating fewer residual water and solvent species retained within the pore channels. As the temperature was further elevated to 300 °C, the TG curve continued to show a downward trend, which is ascribed to the onset of decomposition of silicon methyl groups (Si-CH_3_) on the aerogel surface, generating silanol groups (Si-OH) and consequently resulting in mass loss.

The thermal conductivity of silica aerogel serves as a critical indicator for evaluating its thermal insulation performance, directly reflecting the material’s intrinsic insulating capability. For silica aerogel to function effectively as a high-performance thermal insulator, its thermal conductivity must be maintained at an as-low-as-possible level. Ultimately, the optimal preparation conditions were identified as follows: CO_2_ pressure of 0.3 MPa, sodium silicate concentration of 90 g/L, modification solution ratio of 1:10, and a stepwise drying program of 60 °C→90 °C→120 °C with 2 h at each stage. Under these optimized conditions, the aerogel samples exhibited the following properties: a density of 0.15 g/cm^3^, a specific surface area of 531.4 m^2^/g, a thermal conductivity as low as 0.018 W/(m·K), a pure water contact angle approaching 145°, and favorable thermal stability up to 300 °C.

### 3.3. Characterization and Performance Testing of Aerogel Coatings

**Figure 5:**
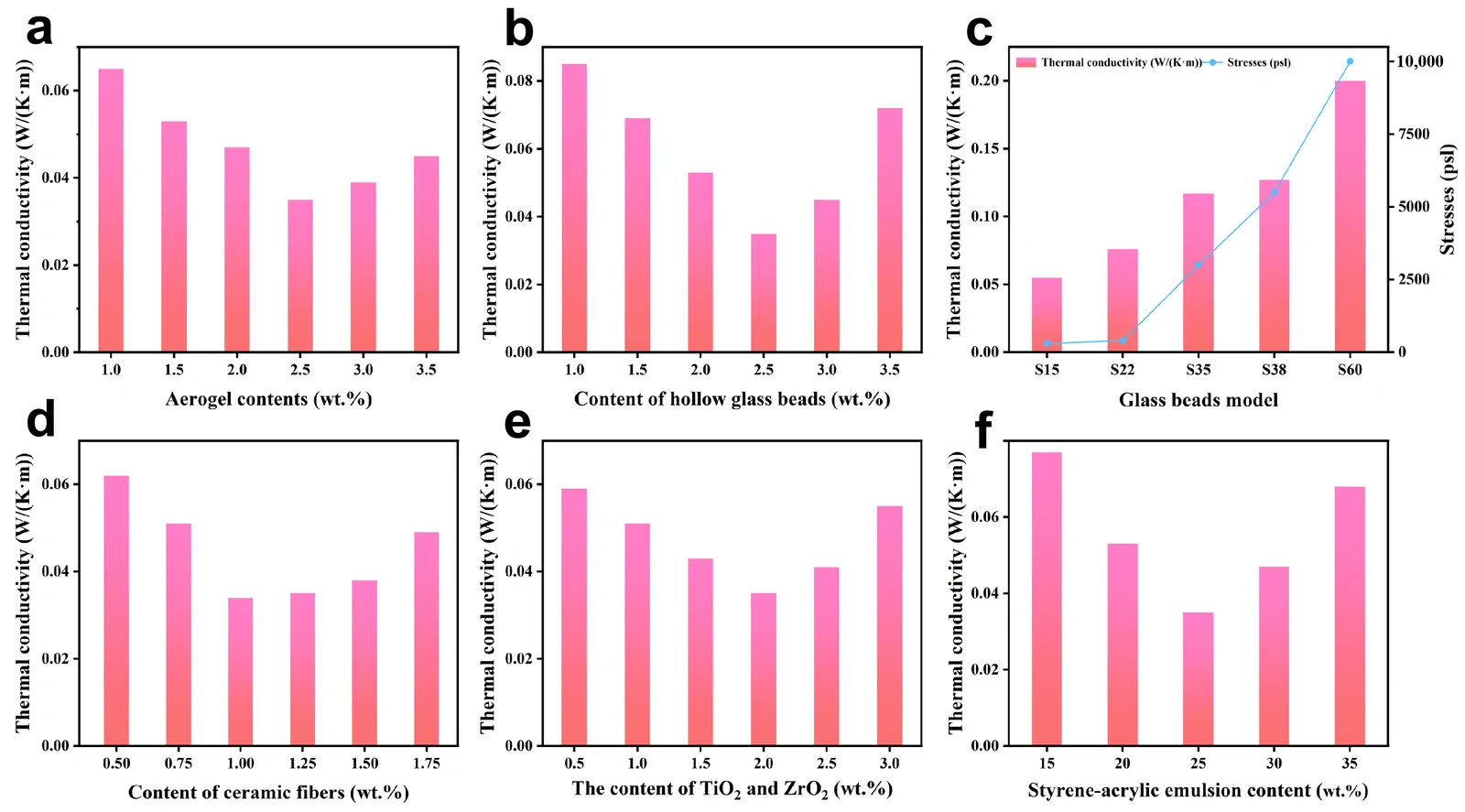
Comparison of thermal conductivity of thermal-insulating coatings prepared under different conditions: (**a**) silica aerogel loading, (**b**) hollow glass microsphere (HGM) loading, (**c**) HGM types, (**d**) aluminum silicate salt loading, (**e**) TiO_2_ and ZrO_2_ loading, and (**f**) styrene–acrylic emulsion loading.

The thermal conductivity of the coating serves as a key indicator for evaluating its thermal insulation performance, as it is directly correlated with the efficiency of heat conduction through the coating [40]. To elucidate the specific effects of different preparation conditions on the thermal insulation performance of the coating, thermal conductivity measurements were conducted on the thermal-insulating coatings prepared under varying conditions. Initially, the thermal conductivity of coatings formulated with different silica aerogel loadings was tested. As shown in Figure 5a, with increasing aerogel content, the thermal conductivity of the thermal-insulating coating exhibited an initial decrease followed by a subsequent increase, reaching its minimum value of 0.035 W/(m·K) at an aerogel loading of 2.5 wt.%. This improvement can be attributed to the intrinsically low thermal conductivity of silica aerogel itself (ranging from 0.016 to 0.020 W/(m·K)), which effectively enhances the thermal insulation capability of the coating. However, when the aerogel loading exceeded 2.5 wt.%, homogeneous mixing between the aerogel and other components became difficult during stirring and blending, resulting in an elevation of the coating’s thermal conductivity. Accordingly, the optimal aerogel loading was determined to be 2.5 wt.%. Figure 5b investigates the effect of hollow glass microsphere (HGM) loading on the thermal conductivity of the coating. When the HGM loading reached 2.5 wt.%, the coating exhibited its minimum thermal conductivity, corresponding to optimal thermal insulation performance. This beneficial effect is primarily attributed to the thin-walled and hollow structure of the hollow glass microspheres. Such a structure, which encloses internal gases with inherently low thermal conductivity, effectively reduces heat conduction efficiency. Meanwhile, the closed-cell configuration of the microspheres impedes thermal convection. The incorporation of a moderate amount of hollow glass microspheres also enables them to fill the internal voids within the coating, thereby retarding heat transfer and further enhancing the thermal insulation performance. However, when the HGM loading was increased beyond this level, insufficient mixing among the components occurred, leading to a deterioration in thermal insulation performance. Therefore, the optimal HGM loading in the coating formulation was determined to be 2.5 wt.%. Different types of hollow glass microspheres exhibit varying thermal insulation effects. During the selection process, two key factors, thermal conductivity and pressure resistance, must be taken into consideration. As shown in Figure 5c, the thermal conductivity of different HGM grades exhibits a positive correlation with their pressure resistance. This implies that hollow glass microspheres with lower thermal conductivity tend to withstand lower pressures. Ideally, it is desirable to select hollow glass microspheres that combine low thermal conductivity with adequate pressure resistance so as to ensure that they remain intact during the mixing and stirring process, thereby maintaining satisfactory thermal insulation performance. Based on a comprehensive consideration of both thermal insulation performance and pressure resistance, the S38 type of hollow glass microspheres was ultimately selected.

Figure 5d compares the thermal conductivity of thermal-insulating coatings prepared with different additions of aluminum silicate salt [41]. When the aluminum silicate salt loading reached 1 wt.%, the thermal conductivity of the coating decreased to its minimum value. This result can likely be attributed to the fact that an appropriate amount of aluminum silicate salt can effectively fill the micro-voids within the coating, and its intrinsically low thermal conductivity significantly reduces heat transfer efficiency. Furthermore, as a fibrous material, aluminum silicate salt also possesses favorable mechanical properties, which can effectively support the internal structure of the aerogel-based coating, mitigating the aggregation of silica aerogel particles and the breakage of hollow glass microspheres that may occur during the mixing and stirring process, thereby ensuring a low thermal conductivity of the coating. As functional fillers in the coating formulation, TiO_2_ and ZrO_2_ also exert a significant influence on the thermal insulation performance of the coating, depending on their respective loadings [42]. As shown in Figure 5e, with increasing TiO_2_ and ZrO_2_ contents, the thermal conductivity of the coating exhibited an initial decrease followed by a subsequent increase. When the combined loading of TiO_2_ and ZrO_2_ reached 2.0 wt.%, the coating achieved its minimum thermal conductivity. This improvement can be attributed to the fact that an appropriate amount of TiO_2_ and ZrO_2_ effectively reflects and scatters incident thermal radiation, thereby reducing heat absorption and transmission. Accordingly, the optimal loading of TiO_2_ and ZrO_2_ was determined to be 2.0 wt.%. Styrene–acrylic emulsion serves as a key film-forming component in the coating formulation. Figure 5f investigates the effect of styrene–acrylic emulsion loading on the performance of the thermal-insulating coating. When the styrene–acrylic emulsion loading reached 25.0 wt.%, the thermal conductivity of the coating decreased to its minimum value, indicating that the thermal insulation performance of the coating reached an optimal state. This improvement can be attributed to the fact that an appropriate amount of styrene–acrylic emulsion exhibits good compatibility with various fillers, facilitating uniform dispersion among the coating components and the formation of a more stable hierarchical pore structure. Simultaneously, it enables the formation of a dense and homogeneous film layer, which effectively impedes external heat transfer, thereby reducing the thermal conductivity of the coating. Accordingly, the optimal loading of styrene–acrylic emulsion was determined to be 25.0 wt.%.

**Figure 6:**
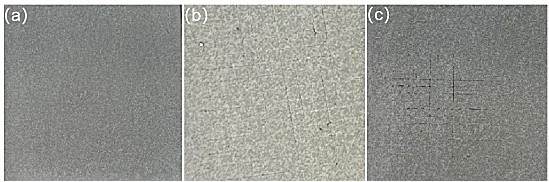
Silica aerogel thermal insulation coating adhesion performance test images: (**a**) before cross-cut test; (**b**) during cross-cut test; (**c**) after cross-cut test.

The adhesion of the optimized coating on stone boards was evaluated by the cross-cut method. The coating exhibited an adhesion grade of 0, with 0% of the coating remaining after tape peeling (Figure 6), indicating good adhesion to the substrate and satisfactory mechanical integrity for architectural decoration applications.

**Figure 7:**
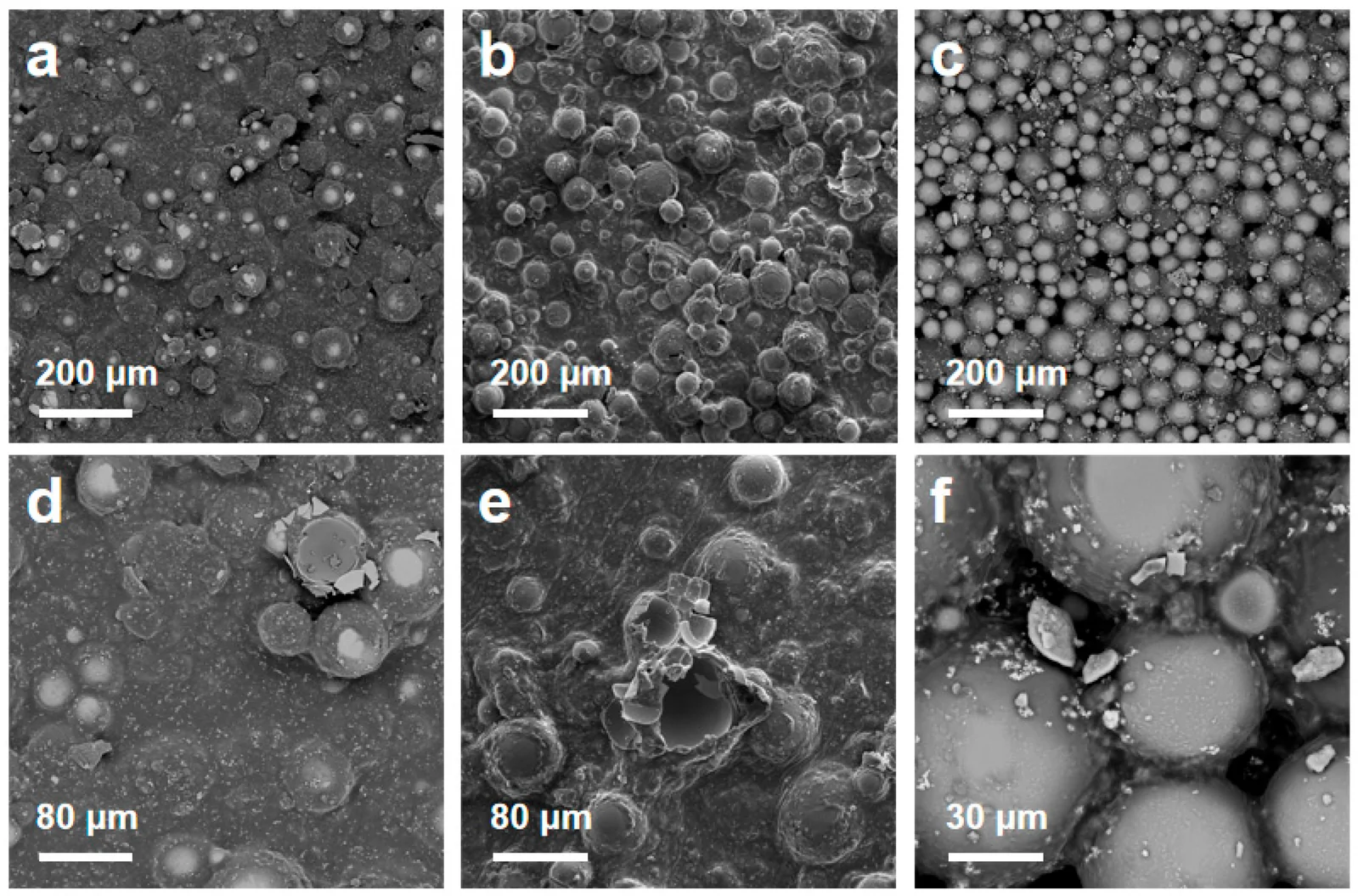
SEM images of thermal-insulating coatings with different aerogel-to-HGM mass ratios: (**a**–**c**) low magnification and (**d**–**f**) high magnification for ratios of 4:1, 1:4, and 1:1, respectively.

The microstructure of the thermal-insulating coating was investigated by scanning electron microscopy (SEM). Figure 7a–c present the micromorphological structures of thermal-insulating coatings prepared with different proportions of insulation fillers (silica aerogel and hollow glass microspheres). It can be observed from the images that the coatings exhibit a uniform and compact microstructural feature, wherein silica aerogel particles and hollow glass microspheres are evenly dispersed and effectively embedded within the coating matrix. The internal structure of the coating is characterized by a porous architecture, with these pores uniformly distributed and interwoven into an intricate network. Figure 7a,d show SEM images of the thermal-insulating coating with an aerogel-to-hollow-glass-microsphere mass ratio of 4:1, revealing that the hollow glass microspheres are largely submerged within the coating matrix, failing to form an effective thermal-insulating network. Figure 7b,e present SEM images of the coating with a mass ratio of 1:4, where numerous hollow glass microspheres are observed to be free-floating on the coating surface. This is attributed to the excessive amount of hollow glass microspheres, which cannot be fully immobilized by the emulsion, consequently leading to an elevated thermal conductivity. In contrast, Figure 7c displays the SEM image of the coating with a mass ratio of 1:1, revealing the formation of a hierarchical pore network structure, with hollow glass microspheres serving as the primary framework and silica aerogel particles penetrating into the emulsion. Figure 7f clearly demonstrates that the silica aerogel particles are uniformly dispersed within the emulsion, tightly bridging the hollow glass microspheres. This hierarchical pore structure, combining micron-scale pores (from hollow glass microspheres) and nanoscale pores (from silica aerogel), not only endows the coating with a high porosity but also significantly disrupts heat transfer pathways, thereby substantially enhancing the thermal insulation performance of the coating. Based on the micromorphological analysis of the thermal-insulating coating, it can be concluded that at an aerogel-to-hollow-glass-microsphere mass ratio of 1:1, the coating exhibits a uniformly dispersed structure and intimate particle interconnections at the microscopic level, which collectively determine its favorable thermal insulation and mechanical properties.

**Figure 8:**
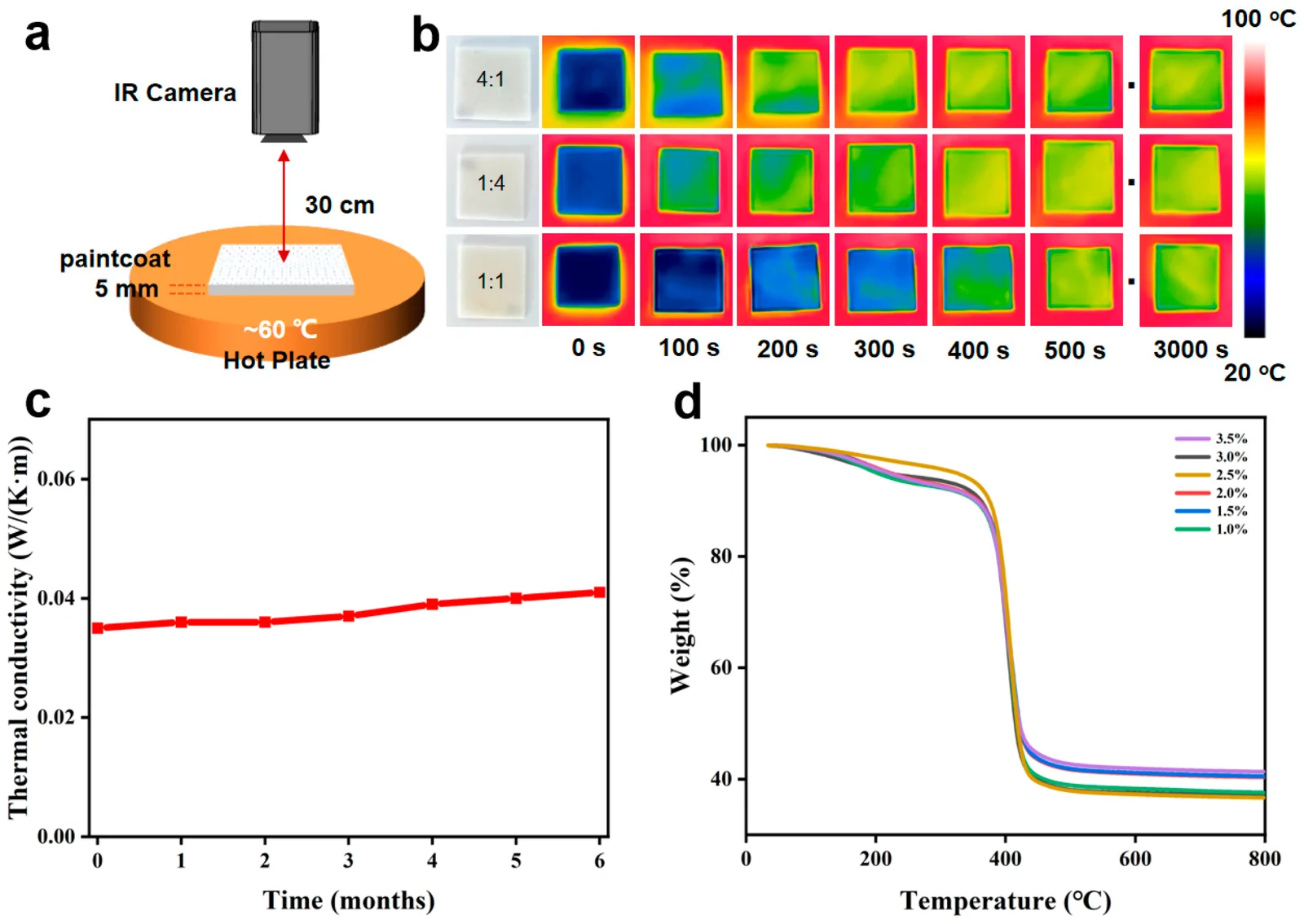
Thermal insulation performance and stability of aerogel-based coatings: (**a**) schematic diagram of the thermal imaging apparatus, (**b**) backside temperature evolution over time for coatings with different aerogel-to-HGM mass ratios, (**c**) thermal conductivity variation of the coating after six months of natural environmental exposure, and (**d**) thermogravimetric (TG) curves of the coating under heating.

To more intuitively demonstrate the performance of the aerogel thermal-insulating coating and the synergistic thermal insulation effect of the hierarchical pore structure constructed by the aerogel and hollow glass microspheres, a thermal imaging apparatus was set up as shown in Figure 8a. The heating plate was maintained at a constant temperature of 60 °C, and an infrared thermal imager was positioned 30 cm above the plate. Coating slices with a thickness of 5.0 mm were placed on the heating plate, and thermal images were recorded every 100 s. As shown in Figure 8b, the backside temperature of all coatings gradually increased and reached a steady state within 3000 s, with steady state defined as a temperature variation of less than 0.5 °C over 5 min. When the aerogel-to-HGM mass ratios were 4:1, 1:4, and 1:1, the final steady-state backside temperatures were 36 °C, 32 °C, and 27 °C, respectively. The 1:1 coating exhibited the lowest steady-state backside temperature, confirming that the hierarchical pore structure constructed by the aerogel and hollow glass microspheres is more favorable for enhancing the thermal-insulating performance of the coating. In practical applications, to ensure that buildings or equipment can continuously achieve energy-saving effects, thermal-insulating coatings must maintain the stability of their thermal insulation performance over prolonged service. Figure 8c presents the variation in thermal conductivity of the thermal-insulating coating under natural environmental conditions. The coating specimens were exposed on a south-facing exterior wall in Hangzhou, China, at a height of approximately 10 m, for six months. The average temperature and relative humidity during exposure were 21.8 °C and 74.5%, respectively. The thermal conductivity was measured by the same steady-state heat flow meter method under identical conditions before and after exposure. The thermal conductivity was 0.035 W/(m·K) before exposure and 0.041 W/(m·K) after six months. These results demonstrate satisfactory long-term thermal-insulating stability under natural environmental conditions. Concurrently, the thermal stability of the coating at elevated temperatures was also evaluated (Figure 8d), employing thermogravimetric analysis (TGA) to monitor the mass changes of the coating during heating. The results revealed that all prepared coating samples exhibited favorable temperature resistance during the initial heating stage. Prior to 350 °C, the TG curves of the coatings showed a gradual declining trend, which is attributed to the volatilization of residual moisture and other solvents within the coating. Upon exceeding 350 °C, however, the TG curves exhibited a pronounced downward trend. Therefore, within the operating temperature range below 350 °C, the thermal-insulating coating demonstrates outstanding thermal stability, rendering it suitable for application in certain relatively high-temperature environments.

To evaluate the performance of the present coating in the context of existing literature, Table 2 compares the thermal conductivity and key properties of the present coating with those reported in representative studies. The present coating achieves a thermal conductivity of 0.035 W/(m·K), which is lower than that of the aerogel/HGM coating reported by Di et al. (0.050 W/(m·K)) [31] and comparable to or better than that of other aerogel-based thermal-insulating coatings [4,24,25]. This improvement is attributed to the synergistic effect between the macropores of the hollow glass microspheres and the nanopores of the silica aerogel, which effectively suppresses both solid heat conduction and internal convection.

## 4. Conclusions

In summary, a novel thermal-insulating composite coating based on silica aerogel and hollow glass microspheres was successfully fabricated via water dispersion technology. The optimal aerogel preparation conditions were established as CO_2_ pressure of 0.3 MPa, sodium silicate concentration of 90 g/L, TMCS/n-hexane ratio of 1:10, and stepwise drying at 60–120 °C, yielding an aerogel with a density of 0.15 g/cm^3^, specific surface area of 531.4 m^2^/g, and thermal conductivity of 0.018 W/(m·K). The optimized coating formulation achieved a minimum thermal conductivity of 0.035 W/(m·K), attributed to the synergistic insulation effect between the macropores of hollow glass microspheres and the nanopores of silica aerogel. At an aerogel-to-HGM mass ratio of 1:1, the coating exhibited a well-developed hierarchical pore structure that effectively suppressed heat conduction and convection, as confirmed by SEM and thermal imaging analyses. The coating also demonstrated excellent long-term stability, with thermal conductivity remaining virtually unchanged after six months of natural exposure and maintaining structural integrity up to 350 °C. This work provides a feasible and scalable strategy for developing high-performance, lightweight thermal-insulating coatings for architectural decoration, and also offers reference value for some industrial thermal-insulation applications.

## Figures and Tables

**Table 1:** The specific surface area data.

CO_2_ Pressure	Specific Surface Area
0.1 MPa	330.9 m^2^/g
0.2 MPa	450.3 m^2^/g
0.3 MPa	531.4 m^2^/g
0.4 MPa	433.2 m^2^/g

**Table 2:** Performance comparison of silica aerogel-based waterborne thermal-insulating architectural coatings.

Reference	Fillers System	Binder	Preparation Method	Minimum Thermal Conductivity (W·m^−1^·K^−1^)
This work	Silica aerogel + HGM (mass ratio 1:1)	Styrene-acrylic emulsion	Mechanical stirring dispersion	0.035
Di et al. [31]	Silica aerogel + HGM (mass ratio 1:1)	Water-borne acrylic resin	Dispersion blending	0.050
He et al. [4]	Hollow glass microspheres and multi-component inorganic fillers	Styrene-acrylic emulsion	Mechanical mixing	Not reported
An et al. [24]	Mesoporous silica aerogel + TiO_2_	Acrylic water-based paint	Dispersion	0.042
Caputo et al. [25]	Bilayer silica aerogel composite	Water-based acrylic	Two-step coating	0.047

## Data Availability

The original contributions presented in this study are included in the article. Further inquiries can be directed to the corresponding author.
